# Spatial modelling of topsoil properties in Romania using geostatistical methods and machine learning

**DOI:** 10.1371/journal.pone.0289286

**Published:** 2023-08-23

**Authors:** Cristian Valeriu Patriche, Bogdan Roşca, Radu Gabriel Pîrnău, Ionuţ Vasiliniuc

**Affiliations:** 1 Geographic Research Center, Romanian Academy, Iaşi Branch, Iaşi, Romania; 2 Department of Geography, Faculty of Geography and Geology, “Alexandru Ioan Cuza” University of Iaşi, Iaşi, Romania; Universiti Teknologi Malaysia, MALAYSIA

## Abstract

Various research topics from the field of soil science or agriculture require digital maps of soil properties as input data. Such maps can be achieved by digital soil mapping (DSM) techniques which have developed consistently during the last decades. Our research focuses on the application of geostatistical methods (including ordinary kriging, regression-kriging and geographically weighted regression) and machine learning algorithms to produce high resolution digital maps of topsoil properties in Romania. Six continuous predictors were considered in our study (digital elevation model, topographic wetness index, normalized difference vegetation index, slope, latitude and longitude). A tolerance test was performed to ensure that all predictors can be used for the purpose of digital soil mapping. The input soil data was extracted from the LUCAS database and includes 7 chemical properties (pH, electrical conductivity, calcium carbonate, organic carbon, N, P, K) and the particle-size fractions (sand, silt, clay). The spatial autocorrelation is higher for pH, organic carbon and calcium carbonate, as indicated by the partial sill / nugget ratio of semivariograms, meaning that these properties are more predictable than the others by kriging interpolation. The optimal DSM method was selected by independent sample validation, using resampled statistics from 100 samples randomly extracted from the validation dataset. Also, an additional independent sample of soil profiles, comprising legacy soil data, and the 200k Romania soil map were used for a supplementary validation. The results show that machine learning and regression-kriging are the optimal methods in most cases. Among the machine learning tested algorithms, the best performance is associated with Support Vector Machines and Random Forests methods. The geographically weighted regression is also among the optimum methods for pH and calcium carbonates spatial prediction. Good predictions were achieved for pH (R^2^ of 0.417–0.469, depending on the method), organic carbon (R^2^ of 0.302–0.443), calcium carbonates (R^2^ of 0.300–0.330) and moderate predictions for electric conductivity, total nitrogen, silt and sand (R^2^ of 0.155–0.331), while the lowest prediction characterizes the phosphorous content (R^2^ of 0.015–0.044). LUCAS proved to be a reliable and useful soil database and the achieved spatial distributions of soil properties can be further used for national and regional soil studies.

## 1. Introduction

Digital soil mapping (DSM) techniques can help achieve accurate maps of soil properties, which are further needed for reliable assessments of soil quality in relation to different land uses. These techniques have developed notably during the last decades, enhanced by the development of geographical information systems (GIS) software, statistical tools and computer technology [1]. Nowadays, there is a wide variety of statistical methods and software which can be used to produce digital maps of soil properties. Currently, DSM techniques are being applied from local to national [2, 3], continental [4, 5] and even global scales [6].

DSM can be successfully used to derive digital maps of both qualitative (soil taxonomic units, soil horizons) and quantitative (various chemical, physical and biological variables) soil properties, as well as to achieve data on pedogenetic processes and to discover new knowledge on soil processes [7]. In the European context, DSM has been used in several studies to predict the occurrence of soil taxonomic units: in Germany (Rhineland-Palatinate area) using artificial neural networks [8], in Hungary (forest environment) using discriminant and classification trees [9], in Germany (Bavarian forest soils) using random forest [10], in Romania (at watershed scale) using logistic regression and fuzzy techniques [11], in Denmark (at country scale) using boosted-decision tree method [12], in Cyprus using random forest [13]. Much more numerous are the studies applying DSM techniques for quantitative soil variables predictions. Soil organic carbon is a parameter of special interest due to its implications in soil fertility and climate change and has been approached in many studies [14]. [15] used geostatistical and machine learning techniques to predict topsoil organic carbon in Sicily (Italy); [16] applied partial least square regression, multiple linear regression and principal component regression in Galicia (Spain); [17] used multiple linear regression, boosted regression trees, artificial neural networks and least-squares support vector machines to estimate top- and subsoil organic carbon stocks in nature reserves in Belgium. Other studies analyzed different or multiple soil properties: [18] focused on modelling soil depth in France; [19] used geostatistical and machine learning techniques and large datasets of covariables to predict several chemical and physical soil properties in areas from Switzerland; [20] used quantile regression forests and regression kriging to predict pH, organic carbon and clay content in the Mediterranean French region.

Digital soil mapping in Romania is still a less approached subject. The only country-wide GIS soil map of Romania is in vector format and it was produced within SIGSTAR-200 project [21] by the digitalization of the soil map units from the analogue 200k scale soil map of Romania [22] and the creation of an attribute database comprising various supplementary information on the soil map units. This digital map was used to derive the topsoil organic carbon content at 500m resolution, using pedotransfer rules [23]. However, its high degree of generalization and the fact that it only includes qualitative parameters within the attribute database makes it unsuitable for a proper evaluations of terrain suitability for crops. Other studies focused on comparative applications of DSM techniques in different regions of the country. [24] used kriging and regression-kriging to derive quantitative soil parameters and logistic regression–kriging to derive occurrence spatial probabilities for soil horizons in a part of Iaşi County; [11] tested the used of logistic regression and fuzzy techniques for the prediction of qualitative soil variables; [25] applied comparatively several geostatistical methods including ordinary kriging, geographically weighted regression, regression-kriging to map soil quantitative properties; [26] investigated the effect of scale and resolution on DSM; [27] used regression-kriging to map soil heavy metals in Cluj County; [28] explored the potential of land-surface segmentation technique to create predictors for DSM of qualitative soil parameters (eg. soil taxonomic units, textural classes); [29] used RK and OK to interpolate pXRF-derived soil chemical data in an archaeological site.Among DSM methods, kriging was one of the first to be used for the interpolation of soil properties in the 80’s [30, 31]. It has developed ever since and now includes several approaches, some simpler (ordinary kriging, simple kriging), other more complex (co-kriging, universal kriging, indicator kriging, probability kriging, kriging with external drift) [32].

In parallel, regression analysis has been used to correlate quantitative soil properties and terrain variables [33]. Coupled with kriging for residuals interpolation, the hybrid regression-kriging method became a powerful digital mapping technique [34–36].

Machine learning (ML) is a more recent approach, the first applications for DSM occurring in the 90’s [37]. It includes a variety of non-linear algorithms originally used for data mining. For DSM purposes, algorithms based on regression and classification, such as *random forest* [38], which is used in our study among other methods, are the most widespread [39–41]. The approach based on ML has the advantage of not making any assumptions regarding data distribution (as it is the case for geostatistical methods) and of handling a large number of cross-correlated predictors. On the other hand, a disadvantage is that the models behind the prediction are very complex and can’t be readily used to explain the prediction [42].

LUCAS (Land Use and Coverage Area frame Survey) (https://esdac.jrc.ec.europa.eu/content/lucas2015-topsoil-data) project began in 2000 following a decision of the European Commission, with the purpose of creating a harmonized soil database across the EU [43]. By repeating the data collection, changes in soil properties can be detected. This database has been successfully used at continental scale to estimate soil erodibility [44], organic carbon [23, 45, 46], copper distribution [47] or other chemical parameters [48, 49]. Several studies also applied LUCAS at country level, eg. to derive soil organic carbon for Belgium and Luxembourg [50], to analyze the evolution of phosphorous content in agricultural soils of Hungary [51]. Some European scale studies regarding the mapping of soil properties through DSM techniques included the Romanian territory as well. [52] modelled the European soil organic carbon stocks under current and future climate and land cover conditions using the regression-kriging approach. [49] used LUCAS 2009/2012 topsoil data to derive maps of 8 chemical parameters for the EU.

The goals of our study are to test several spatial interpolation methods, choose the optimal one through independent validation and to provide high resolution maps of topsoil parameters based on LUCAS database, potentially useful for regional and local agricultural land evaluation. It is the first time that digital mapping techniques, including geostatistical methods and machine learning, are applied for the entire Romanian territory. In relation to these goals, the research questions we address in our study are the following: (i) which is the optimal DSM method for the analyzed soil properties; (ii) how accurate are the resulting spatial models; (iii) how reliable is the LUCAS database for national and regional soil assessments. Compared to the European scale studies which have included the Romanian territory, our study brings more insight for several reasons: it is focused only on Romania; it applies several DSM methods in order to choose the optimal one; it uses more recent LUCAS data (2015 for chemical properties) and provides spatial models for more soil variables (in addition to pH, CaCO_3_, N, P, K, we mapped organic carbon content, electrical conductivity and particle-size variables–clay, silt, sand); it provides higher resolution maps (100x100 m).

## 2. Materials and methods

### 2.1. Study area

Romania is a medium-size European country (about 237,000 km^2^) situated in the central-eastern part of the continent. It has a temperate continental climate with mean annual temperature of about 9.5°C, ranging from <0°C on the highest mountain peaks to >11°C in the southern and south-eastern plain and plateau regions. The precipitations increase with elevation and from east to west, with a mean annual value of about 635 mm yr^-1^ and a value range from <400 mm yr^-1^ in eastern Dobrogea and >1000 mm yr^-1^ on the highest mountain peaks [53]. The surface lithology is characterized by the dominance of loess and loess-like formations in the plain areas, lime and sandstones in the plateau and hilly areas and hard metamorphic, igneous and sedimentary rocks in the mountain areas. Land use in dominated by agriculture in the lower plain, hilly and plateau areas (57% of the country).

**Fig 1:**
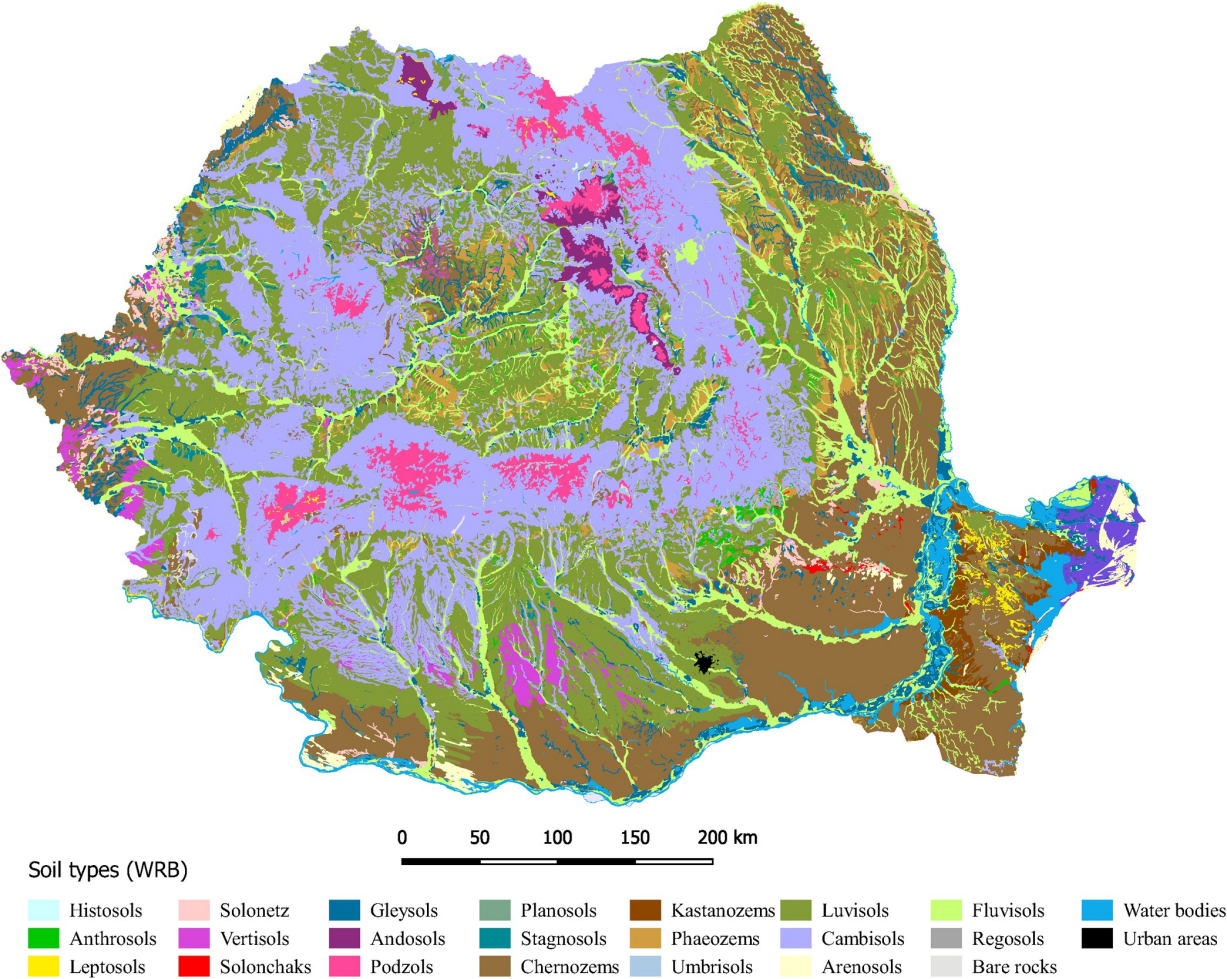
Soil map of Romania [22, 23].

A general overview regarding the soil distribution in Romania is shown in Fig 1. The main characteristic of the Romanian soil cover is the concentric distribution of the major soil zones around the Carpathian Mountains. The mountain area is covered mainly by Cambisols, Podzols and Umbrisols, with a consistent extent of Andosols in the volcanic area from Eastern Carpathians. The hilly area and the large plateaus surrounding the mountains are dominated by Luvisols. They gradually turn into Phaeozems followed by the broad belt of Chernozems which occurs in the peripheral low plains. Fluvisols are present in the large river valleys, usually accompanied by Gleysols and saline soils. The relatively equal percentages of Cambisols (24.8%), Luvisols (24.2%) and Chernozems (22.1%) are consistent with the distribution of major landforms (mountains, plateaus / hills and plains) across Romania.

### 2.2. LUCAS soil database

The LUCAS (Land Use and Coverage Area frame Survey) project started in 2001, following the 1445/2000 decision of the European Commission, its target being to continue the monitoring of land use and land cover in the EU, providing information related to the sustainable development of environment, landscapes, agriculture and terrain degradation. In 2006, the survey changed its sampling methodology and broadened its focus and frequency. Since 2009 the sampling has included topsoil surveys, in order to construct a robust and standardized database regarding the soil cover. The purpose implied various applications of the dataset, including of modelling soil properties, evaluating soil implication in climate change, nutrient cycles, agriculture or comparing the quality of national / regional soil inventories. The same year, the covered area has been expanded to most of the EU states, Bulgaria and Romania being sampled in 2012. A description of LUCAS 2012 database for Romania and Bulgaria is provided by [43]. In 2015, sampling was expanded to cover locations at altitudes above 1000 m [54].

In order to establish baseline values of topsoil properties, the sampling points have been selected at similar densities in each country. However, differences occur due to a preference for agricultural land in comparison to grasslands and woodlands, which can limit the quality of the statistical extrapolations or the use of the data for soil mapping [48].

The sampling methodology and laboratory analyses are the same since 2009. The methods used for the analysis of physical and chemical properties in topsoil samples are detailed in [55], while detailed description of the sampling efficiency is described by [56].

LUCAS Topsoil Survey is based on the soil parameters that are deemed relevant for agricultural policy, providing an excellent basis to assess the changes in topsoil characteristics across the EU. Therefore, analysis of physical and chemical properties represents the core of the LUCAS Soil survey. Each property has its standard protocol, and all samples are analyzed following the same procedure. This makes the data comparable over time [48, 54]. In this study we analyzed 10 parameters extracted from LUCAS 2012 and 2015 topsoil databases: soil pH, CaCO_3_, organic carbon (OC), total nitrogen (N), phosphorous content (P), extractible potassium content (K), electrical conductivity (EC) and particle-size distribution (clay, silt, sand).

**Fig 2:**
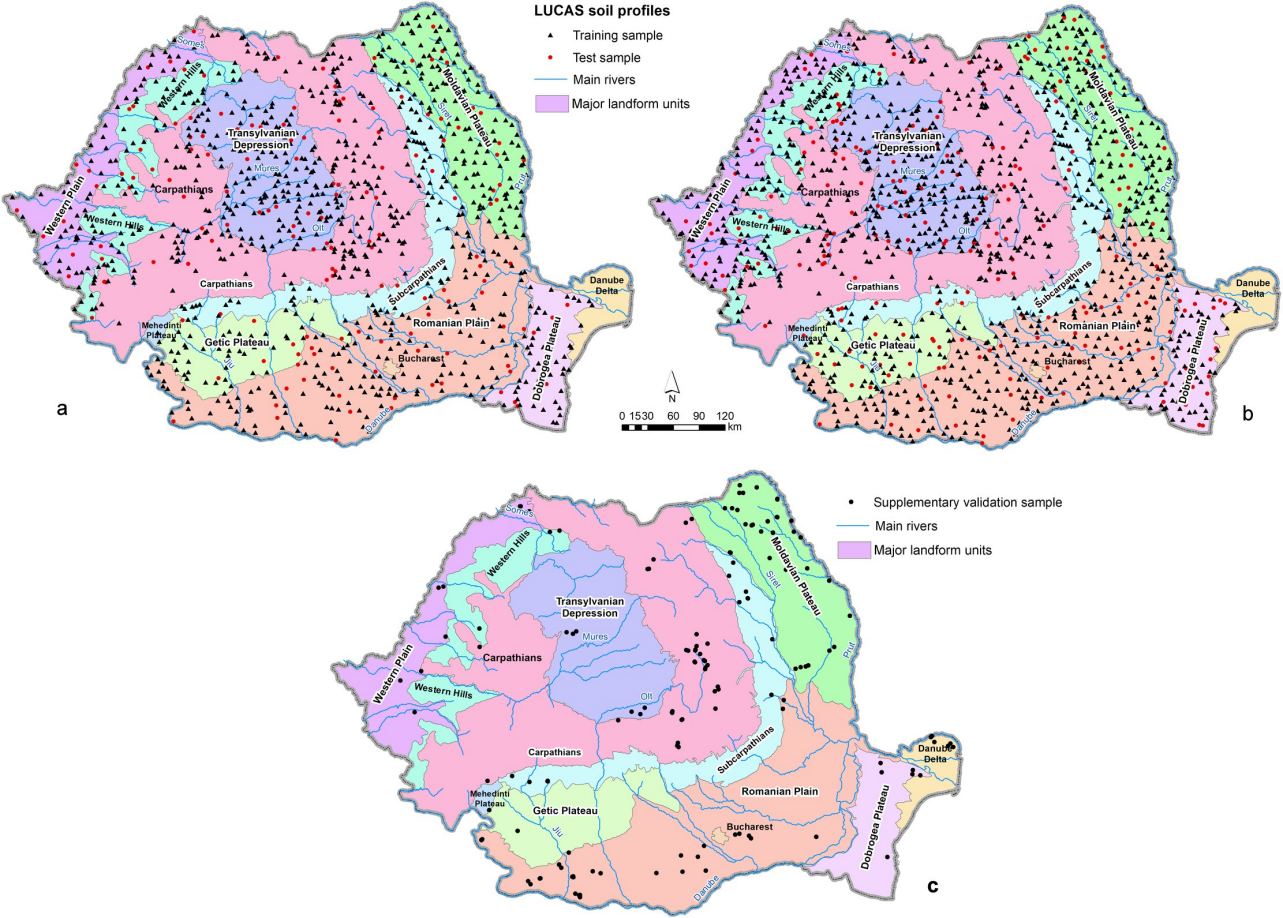
Training and test samples used for interpolation of chemical (a) and particle-size (b) properties (extracted from LUCAS database: https://esdac.jrc.ec.europa.eu/projects/lucas) and the supplementary validation sample (c) [57] overlaying the major landform units of Romania [58].

LUCAS 2012 and 2015 databases were extracted for Romania. The 2015 database, comprising 1083 points, was used to interpolate the chemical soil properties. A random subsample of 160 points (about 15% of the total sample) was extracted and used for independent validation (Fig 2). The 2012 database and the additional points in 2015 database, totaling 1444 points, were used to interpolate the particle-size fractions. A random subsample of 220 points (about 15% of the total sample) was extracted for independent validation.

### 2.3. Predictor variables

**Fig 3:**
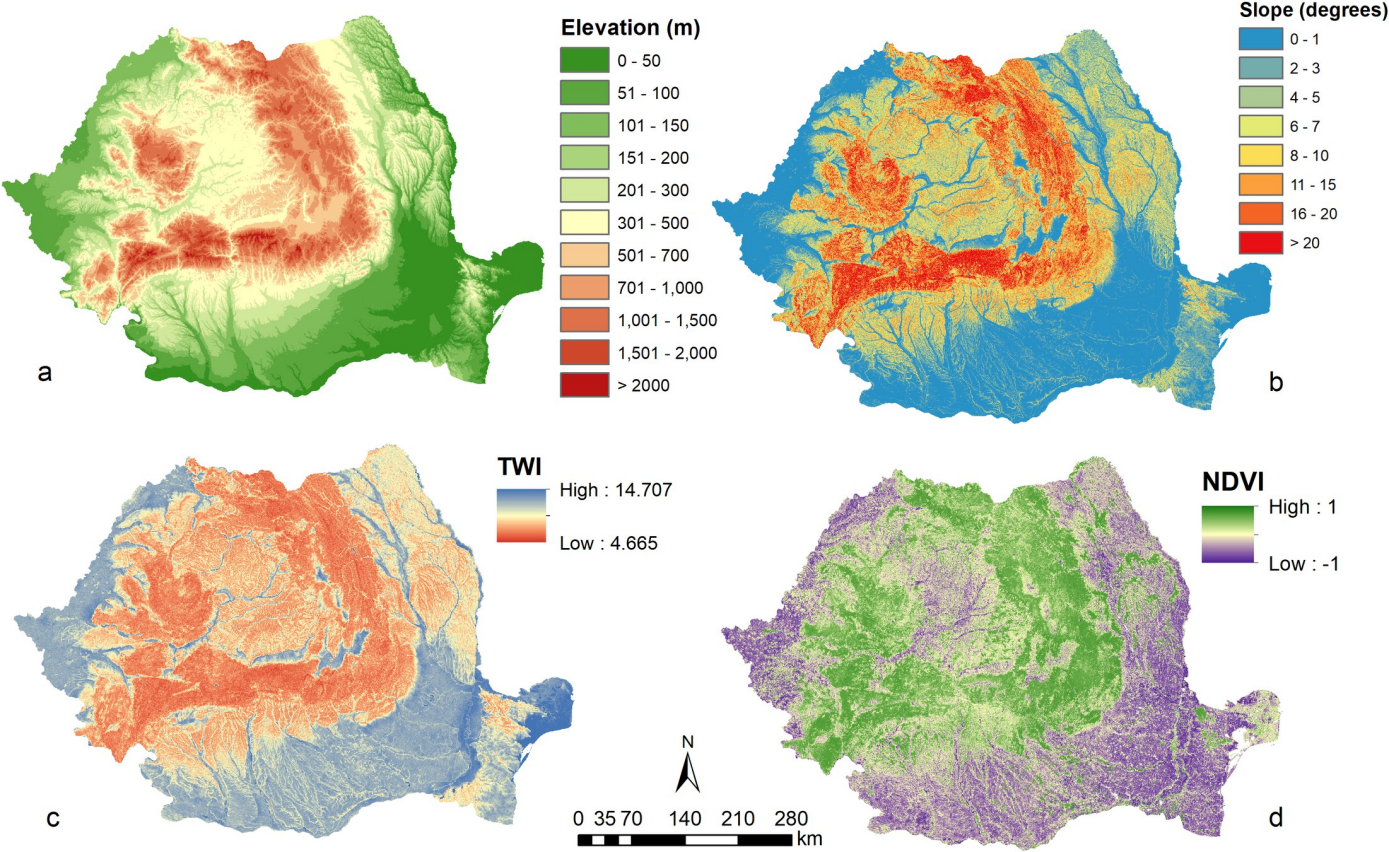
Predictor maps used in interpolation procedures: a–elevation; b–slope; c–topographic wetness index; d–normalized difference vegetation index.

We used 6 predictors in our study as covariates in RK, GWR, GWR-OK and EML methods: digital elevation model (DEM), slope (S), topographic wetness index (TWI), normalized difference vegetation index (NDVI) (Fig 3), latitude (LAT) and longitude (LON). We preferred to use a minimum number of significant predictors, instead of a large predictor dataset, in order to avoid overfitting and multicollinearity problems and to ensure that the achieved models can be explained through the contributions of the factors. Also, a small number of quantitative predictors facilitates model reproduction in different areas, at different scales.

The spatial analysis was performed at 100 m resolution using ArcGIS 10.8 software (ESRI, https://www.esri.com/en-us/arcgis/). The DEM was extracted from EUDEM (https://land.copernicus.eu/imagery-in-situ/eu-dem/eu-dem-v1.1/view) and it was further used to derive slope and topographic wetness index, the latter in SAGA-GIS software (http://www.saga-gis.org/). NDVI was derived from LANDSAT 8 imagery using Google Earth Engine (https://developers.google.com/earth-engine/datasets/catalog/landsat-8). The median values of June-August interval from 2015–2020 were used to compute this predictor. The choice of the summer months for NDVI computation is related to the vegetation development, which is at its peak under the temperate continental conditions of Romania, while the different types of vegetation are best discriminated during summer.

The DEM is probably the most important factor, expressing the altitudinal distribution of soils as effect of climate and vegetation zonation, but also as effect of different lithology (soft, loamy loess-like formations in the lower plain and hilly regions and hard rocks in the mountain areas). Slope influences soil erosion, while topographic wetness index expresses the accumulation potential of the relief for water and sediments. NDVI accounts for the influence of vegetation on soil properties, while latitude and longitude may account for general eastward / northward trends induced by climate variations.

### 2.4. Spatial interpolation methods

Our study compares the outcome of 5 spatial interpolation methods, namely ordinary kriging (OK), regression-kriging (RK), geographically weighted regression (GWR), geographically weighted regression–kriging (GWR-OK) and ensemble machine learning (EML).

*Ordinary kriging (OK)* is a local interpolation method based on the autocorrelation concept [59], according to which the values of a variable are similar in close locations and the dissimilarity increases with the distance between points. The dissimilarity is measured through semivariance, while the chart displaying the semivariances against point distances is called semivariogram and it is used to derive point weights on the basis of which the estimated values are computed. The empirical variogram, as a nonparametric estimator of the variogram of a spatial process, is described by the following equation according to the Matheron estimator:

$$\gamma (h)=\frac{1}{2N(h)}\sum_{i=1}^{N(h)}{\left(Z_{i}-Z_{i+h}\right)}^{2}$$ (1)

where:

- *γ(h)*: semivariance of points separated by distance *h*;
- *Z*_*i*_: variable values in points *x*_*i*_;
- *Z*_*i+h*_: variable values in points situated at distance *h* from points *x*_*i*_;
- *N(h)*: number of points situated at distance *h*.

The kriging estimator at unsampled locations is given by a linear combination of the variable values at known locations:

$$Z^{*}\left(x\right)=\sum_{i=1}^{N}\lambda_{i}Z\left(x_{i}\right)\pm \epsilon$$ (2)

where:

- *Z(x*_*0*_*)*: kriging estimator at unsampled location x;
- *Z(x*_*i*_*)*: variable values in *x*_*i*_ neighboring points;
- λ_i_: weighting coefficients of *x*_*i*_ neighboring points, $\sum_{i=1}^{N}\lambda_{i}=1$;
- ε: standard error.

The weighting coefficients (λ_i_) are determined in such a way as to minimize the estimation variance. Ordinary kriging doesn’t take into account co-variables for interpolation, the predicted values depending only on the neighboring values. Ordinary kriging has been extensively used for mapping soil [30, 31, 60–62]. In our study, ordinary kriging interpolation was applied by using the Geostatistical Analyst module from ArcGIS 10.8 software (ESRI, https://www.esri.com/en-us/arcgis/).

*Regression-kriging (RK)* combines regression analysis with ordinary kriging into a theoretically superior interpolation method [33, 34]. Our study uses global linear regression models to derive spatial trends of topsoil parameters:

$$\hat{Y}=a+\sum_{i=1}^{N}b_{i}\cdot X_{i}\pm \varepsilon$$ (3)

where:

- $\hat{Y}$: predicted value;
- *X*_*i*_: predictors;
- *a*: intercept;
- *b*_*i*_: regression coefficients;
- *ε*: standard error.

The deviations from this trend (residuals) are then interpolated by ordinary kriging, the final spatial model resulting from the addition of spatial trend and residuals. As ordinary kriging, the regression-kriging method has numerous applications for mapping soil parameters [32, 36].

A stepwise integration of predictors into the regression models was applied in order to avoid collinearity and insert only the predictors with significant contributions to the prediction.

*The geographically weighted regression (GWR)* applies local regression models in moving windows with fixed or variable dimensions [63]. Regression models are computed for each location based on the neighboring point values. These points receive different weights according to a gaussian function. The closer a point is to the center of the moving window, the higher is its weight and contribution to the estimated value. In contrast to global regression, GWR has the advantage of rendering local variations. Combining GWR with ordinary kriging of residuals leads to the hybrid *geographically weighted regression–kriging (GWR-OK)* method. GWR and GWR-OK have also been successfully used for digital mapping of soil parameters [25, 64, 65]. In our study, ordinary kriging interpolation was applied by using the Geographically Weighted Regression module from ArcGIS 10.8 software (ESRI, https://www.esri.com/en-us/arcgis/).

*Ensemble machine learning (EML)* as described by [66, 67] is a predictive technique which combines two or more models to improve the prediction accuracy. Compared to individual learners, the use of ensemble learners has several advantages: it improves performance, by combining the output of multiple learners, it ensures robustness, by reducing the extrapolation / overshooting effects, and minimizes bias.

For this study, we used EML implemented in *landmap* package for R [68], which extends the functionality of mlr package [69] and contains the necessary tools and functions for automatic mapping of environmental variables without too much intervention required from the user.

The package has 5 stacked learners implemented by default ("regr.cvglmnet"–Generalized Linear Model with Lasso or Elasticnet Regularization, "regr.ksvm"—Support Vector Machines, "regr.nnet"—Neural Network, "regr.ranger"—Random Forests, "regr.xgboost"—eXtreme Gradient Boosting) and allows the use of oblique coordinates [70] as well as the geographical distance to all as “geographical features” [71, 72]. By default, the *landmap* package allows the user to train a spatial prediction using a SuperLearner algorithm [73] which consists in computing each individual learner by 5-fold cross-validation and determine the meta-learner. Due to the large numbers of points (over 900) we used only oblique coordinates with a list of 28 angles [70] to reduce the artefacts.

The *landmap* package integrates several techniques, allowing an automate application of EML, with minimum user intervention [68]: derivation of geographical distances, computation of principal components based on a covariate dataset, automated filling of gaps, automated fitting of variogram, spatial data overlay, cross-validation model training, fitting of the final EML model.

### 2.5. Validation procedure

For validation purposes, we used randomly selected independent samples which were not included in the model computation stage, representing about 15% of the total samples (160 points for chemical soil properties and 220 points for particle-size fractions). Correlation charts, displaying observed vs predicted values, were computed for each soil parameter and interpolation method, while the selection of the best model was based on the following statistical quality indices:

- *Coefficient of determination (R*^*2*^*)*, which is the squared Pearson correlation coefficient:

$$R^{2}={\left[\frac{\mathrm{cov}(\hat{Y},Y)}{\sigma_{\hat{Y}}\sigma_{y}}\right]}^{2}$$ (4)

where:

 ‐ *COV(X*,*Y)*: covariance of predicted ($\hat{Y}$) and observed (Y) values;
 ‐ *σ*_*X*_, *σ*_*Y*_: standard deviations of predicted ($\hat{Y}$) and observed (Y) values.
  - *Mean absolute error (MAE)*:

$$MAE=\frac{1}{N}\sum_{i=1}^{N}|Y_{i}-{\hat{Y}}_{i}|$$ (5)

where:

 ‐ *Y*_*i*_: observed values;
 ‐ ${\hat{Y}}_{i}$: predicted values;
 ‐ N: validation sample size.
  - *Root mean square error (RMSE)*:

$$RMSE=\frac{1}{N}\sum_{i=1}^{N}{\left(Y_{i}-{\hat{Y}}_{i}\right)}^{2}$$ (6)

In addition, the *mean bias error* was computed in order to evaluate the bias of observed–predicted relationships:

$$ME=\frac{1}{N}\sum_{i=1}^{N}\left(Y_{i}-{\hat{Y}}_{i}\right)$$ (7)

High positive values of ME indicate that the model underestimate the observed values, while low negative ME values indicate that the model tend to overestimate the observed values.

In order to refine the validation stage, the test datasets were randomly resampled using 85% of the data points into 100 samples which were further used to compute average and standard deviation values of the validation parameters (R^2^, MAE, RMSE and ME). Resampling was achieved in Excel environment using *Resampling Stats Addin* module Bruce P. (https://resample.statistics.com/excel/).

A supplementary independent sample was also used for validation, comprising legacy soil data from 148 soil profiles (Fig 2C). This database was compiled from various soil studies conducted in Romania during 1960–2020 period using standard laboratory determinations. The database has been compiled, verified and used for the first time by [57]. We also performed a validation of our quantitative soil parameter maps with the 200k Romanian soil map by computing average parameter values for soil reference groups and interpreting the plausibility of results.

## 3. Results

### 3.1. Descriptive statistics, variable correlations and variography

LUCAS dataset for Romania spans from sea level to about 1500 m altitude and cover slopes up to 29^o^ (Table 1). It is therefore important to keep in mind possible extrapolation errors at higher elevations and on steeper slopes. Among soil properties, CaCO_3_ and P content present the highest spatial variability. We notice that the median value is 0 for CaCO_3_, meaning that more than half of the sample has 0 values for this parameter. P content is characterized by the presence of some very high peak values, exceeding 300 mg kg^-1^. The lowest spatial variability characterizes soil pH, followed by clay and silt contents.

**Table 1:** Descriptive statistics of LUCAS soil properties and predictor variables.

Variable	Min	Max	1st Quartile	Median	3rd Quartile	Mean	Variance	Standard deviation	Variation coefficient
*pH*	3.540	9.630	5.270	6.030	7.410	6.208	1.439	1.199	0.193
*EC (mS m* ^*-1*^ *)*	2.170	265.000	10.535	15.700	22.600	19.125	246.302	15.694	0.820
*OC (g kg* ^*-1*^ *)*	1.400	354.700	14.200	18.600	24.950	23.001	430.408	20.746	0.902
*CaCO*_*3*_ *(g kg*^*-1*^*)*	0.000	486.000	0.000	0.000	3.000	11.513	979.293	31.294	2.717
*P (mg kg* ^*-1*^ *)*	0.000	380.300	6.600	10.600	18.050	17.381	791.992	28.142	1.618
*N (g kg* ^*-1*^ *)*	0.200	31.800	1.600	2.000	2.600	2.309	2.284	1.511	0.654
*K (mg kg* ^*-1*^ *)*	21.300	3750.700	115.350	178.200	263.050	230.039	55826.080	236.275	1.027
*Clay (%)*	0.000	82.000	23.000	41.000	56.000	39.650	394.304	19.857	0.501
*Silt (%)*	1.000	93.000	21.000	30.000	44.000	34.845	360.467	18.986	0.545
*Sand (%)*	0.000	99.000	9.000	21.000	37.000	25.505	411.408	20.283	0.795
*DEM*	1.787	1496.980	110.511	248.430	449.521	336.355	95289.720	308.690	0.917
*SLOPE*	0.038	28.802	0.571	2.804	6.512	4.293	21.952	4.685	1.091
*NDVI*	0.159	0.903	0.417	0.551	0.667	0.549	0.030	0.173	0.316
*TWI*	5.890	13.106	7.948	9.307	11.048	9.440	2.943	1.715	0.182

The Pearson correlation matrix (Table 2) shows that there are significant correlations among the predictor variables, the highest being between TWI and slope (-0.805). However, the two predictors can’t be seen as redundant because they express different, though complementary, sides of terrain reality: slope reflects the erosion potential, while TWI reflects the accumulation potential. The other correlation values are moderate and low. A tolerance test was carried out to ensure that the predictors can be all used in digital mapping procedures. Tolerance values are computed as 1 –R^2^ (XLSTAT Tutorial, https://help.xlstat.com/), where R^2^, for a certain variable, is the determination coefficient of the prediction of all other variables. Tolerance values less than 0.2 [74] or 0.1 [75] indicate the presence of multicollinearity, that is the presence of significant correlations among the predictor variables which may induce wrong interpretations of the variable contributions to the final spatial models. In our situation, the minimum tolerance value is 0.3, indicating that all predictors can be used in prediction models.

**Table 2:** Pearson correlation matrix and tolerance values for predictors.

Variables^*^	*pH*	*EC*	*OC*	*CaCO*_*3*_	*P*	*N*	*K*	*Clay*	*Silt*	*Sand*	*DEM*	*SLOPE*	*NDVI*	*TWI*	*LAT*	*LON*
*pH*	**1**															
*EC*	0.044	**1**														
*OC*	**-0.193**	**0.388**	**1**													
*CaCO*_*3*_	**0.517**	**0.134**	-0.012	**1**												
*P*	0.020	**0.194**	**0.199**	-0.048	**1**											
*N*	**-0.101**	**0.443**	**0.932**	0.031	**0.228**	**1**										
*K*	**0.286**	**0.202**	**0.143**	**0.124**	**0.610**	**0.241**	**1**									
*Clay*	-0.052	**-0.174**	**-0.124**	**-0.244**	0.014	**-0.075**	**0.076**	**1**								
*Silt*	**0.289**	**0.211**	**0.158**	**0.382**	-0.014	**0.186**	**0.157**	**-0.455**	**1**							
*Sand*	**-0.219**	-0.027	-0.027	**-0.119**	-0.001	**-0.101**	**-0.221**	**-0.553**	**-0.490**	**1**						
*DEM*	**-0.534**	**0.267**	**0.449**	**-0.147**	**-0.062**	**0.384**	**-0.188**	**-0.306**	**0.103**	**0.203**	**1**					
*SLOPE*	**-0.282**	**0.175**	**0.279**	-0.015	**-0.080**	**0.243**	**-0.066**	**-0.242**	**0.065**	**0.176**	**0.627**	**1**				
*NDVI*	**-0.379**	**0.183**	**0.290**	**-0.125**	-0.019	**0.281**	**-0.072**	**-0.232**	0.011	**0.218**	**0.563**	**0.479**	**1**			
*TWI*	**0.283**	**-0.110**	**-0.212**	0.053	**0.108**	**-0.183**	**0.072**	**0.175**	0.017	**-0.187**	**-0.605**	**-0.805**	**-0.497**	**1**		
*LAT*	**-0.164**	**0.117**	**0.164**	**-0.103**	0.002	**0.200**	0.056	-0.025	0.043	-0.016	**0.331**	**0.324**	**0.267**	**-0.421**	**1**	
*LON*	**0.320**	-0.023	-0.004	**0.199**	-0.033	0.009	**0.082**	-0.048	**0.249**	**-0.186**	**-0.123**	-0.005	**-0.166**	0.036	**-0.073**	**1**
***Tolerance*** ^**^											*0*.*515*	*0*.*316*	*0*.*633*	*0*.*300*	*0*.*807*	*0*.*956*

^*^ correlation values in bold are different from 0 with a significance level alpha of 0.05

^**^ tolerance values smaller than 0.2 are indicators of multicollinearity

The correlations between soil variables and predictors (Table 2) are generally significant, due to the large sample size, but of low or moderate magnitude. Soil reaction (pH) has the highest number of significant correlations and the highest magnitude, while the silt content seems to be the variable the least correlated with the predictor variables. The correlations among soil variables show, as expected, a close dependency of pH on CaCO_3_ content and a very tight relationship between OC and N. Also, the high correlation between P and K is probably the effect of their origin from the same source, namely the fertilizers.

The total LUCAS samples are characterized by a mean point distance of about 8.5 km for the chemical properties sample and of 6.7 km for the particle size sample, 50% of the points being separated by distances of less than 19–20 km. The mean point densities of the two samples are 4.5 and 6.1 points / 1000 km^2^ respectively, 50% of the territory having densities of more than 8.5–10.0 points / 1000 km^2^.

**Fig 4:**
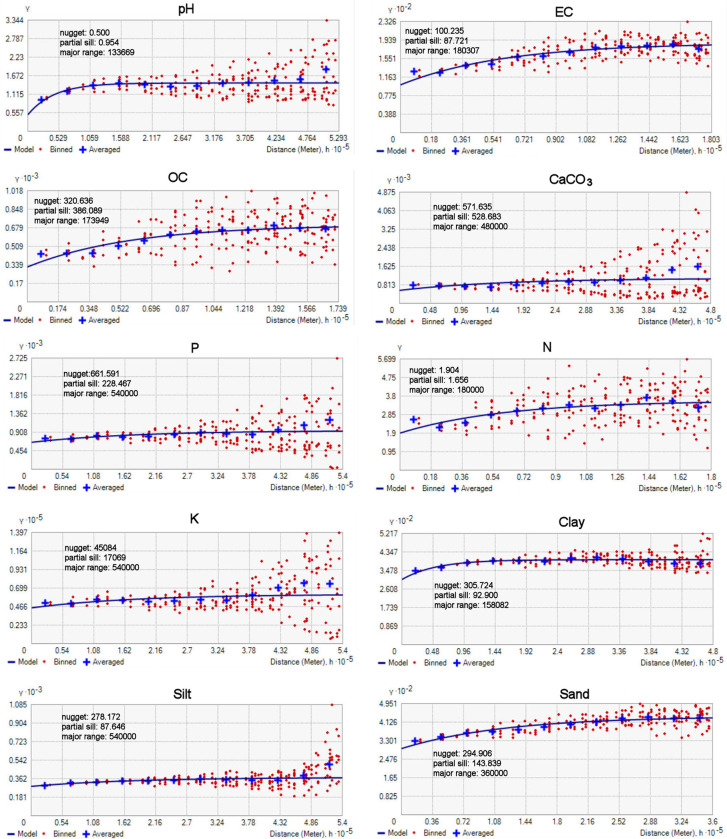
Semivariograms of soil properties.

According to variography (Fig 4), the partial sill / nugget ratio shows that the spatial autocorrelation is higher for pH, OC and CaCO_3_, these properties being therefore more predictable than the others by kriging interpolation, while the spatial autocorrelation is lower for clay, silt and P, in which case the kriging predictions are expected to be more uncertain. Among the available semivariogram models, the exponential type was chosen to fit the experimental semivariogram cloud because it minimized the nugget effect. The lag size is generally around 44–45 km, except for OC, EC and N in which case it is smaller (around 15 km) indicating more localized spatial variations. Anisotropy was not considered in the variography analysis because a single clear data variation direction could not be identified throughout the country due to landscape complexity.

### 3.2. Spatial representativeness of predictor variables

**Fig 5:**
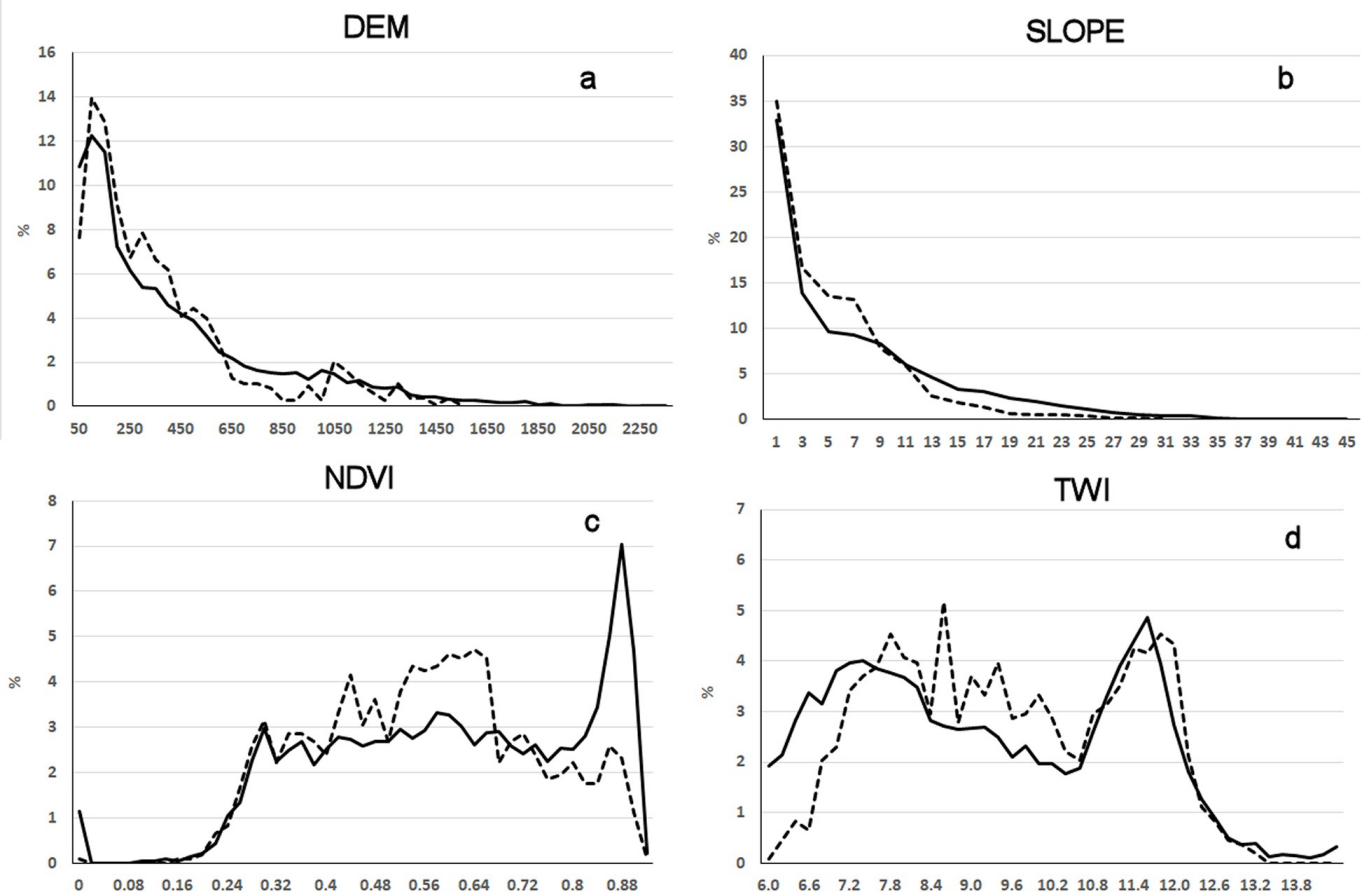
Spatial representativeness of LUCAS 2015 total sample in respect to the main predictors: a–elevation; b–slope; c–topographic wetness index; d–normalized difference vegetation index.

Fig 5 displays the spatial representativeness of the predictors (DEM, SLOPE, NDVI, TWI) by comparing the relative frequencies of classes based on predictors and LUCAS 2015 sample points. We notice a good agreement between the histograms for elevation and slope. Some minor differences indicate slight underrepresentation in the medium elevation domain (700–1000 m) and at higher slopes (> 13^o^). Also, LUCAS 2015 sample doesn’t contain points with elevations higher that 1550 m and with slope values higher than 31^o^, which may induce model extrapolation problems. For NDVI and TWI there are no extrapolation issues, the point sample covering all value classes. However, the comparison of the frequency curves shows some differences related to the underrepresentation of the sample in the high value domain for NDVI (> 0.8) and in the lower value domain for TWI (<7.6).

### 3.3. Spatial models of soil properties

**Fig 6:**
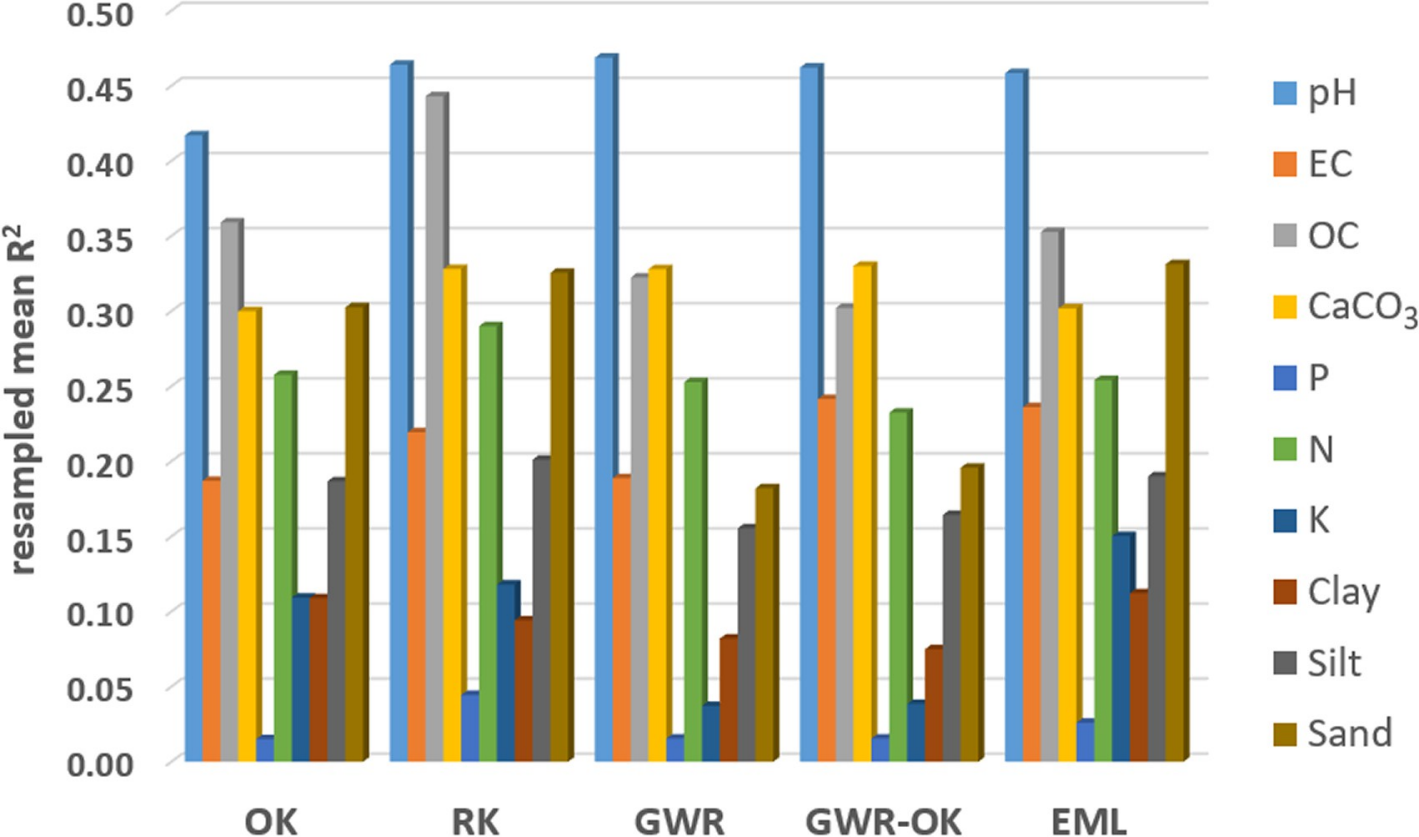
Mean R^2^ values computed for resampled validation datasets.

The coefficients of determination (R^2^) values show that the most predictable soil property is the pH, in the case of which 41–47% of the spatial variance of the validation sample could be accounted for by the computed spatial models (Table 3 and Fig 6). Good performances were also achieved for OC (R^2^ of 0.302–0.443) and CaCO_3_ (R^2^ of 0.300–0.330). The weakest prediction characterizes the phosphorous content (P), for which none of the spatial interpolation methods provided a statistically significant model with the initial settings. Still, in order to provide a map for this soil parameters as well, and considering the fact that the tolerance test showed no multicollinearity, we made an exception and computed the regression model using all predictors, regardless of their contribution to prediction. The model achieved has a low (R^2^ of 0.044) but statistically significant explained variance for the 0.05 significance level. Weak performances were also achieved for K (R^2^ of 0.037–0.150) and clay content (R^2^ of 0.075–0.112). Moderate performance was achieved for the other soil parameters (EC, N, silt, sand), for which the explained variances computed for validation samples are about 20–30%. The overall determination coefficients may seem small, but in the context of soil prediction by digital soil mapping, R^2^ values of 0.3–0.5 are commonly found [36, 61, 76].

**Table 3:** Mean values and standard deviations of quality statistical indices computed for 100 resampled validation datasets^*^.

pH					EC			
*Method* ^**^	*R* ^*2*^	*ME*	*MAE*	*RMSE*	*R* ^*2*^	*ME*	*MAE*	*RMSE*
*OK*	0.417±0.067	0.060±0.087	0.683±0.535	0.953±0.057	0.187±0.085	0.499±0.964	6.899±5.882	11.325±1.861
*RK*	0.464±0.061	0.048±0.077	0.685±0.523	0.918±0.048	0.219±0.086	0.454±0.898	6.924±5.928	11.130±1.809
*GWR*	0.469±0.064	0.006±0.074	0.752±0.585	0.912±0.059	0.189±0.068	-0.325±1.163	8.457±9.116	13.152±1.461
*GWR-OK*	0.462±0.061	0.037±0.078	0.729±0.591	0.916±0.053	0.241±0.064	-19.828±1.197	22.253±13.763	24.540±1.097
*EML*	0.458±0.063	0.450±0.071	0.766±0.630	1.019±0.058	0.236±0.062	-0.044±0.923	7.903±7.670	11.087±1.686
**OC**					**CaCO**_**3**_			
***Method***	***R*** ^***2***^	***ME***	***MAE***	***RMSE***	***R*** ^***2***^	***ME***	***MAE***	***RMSE***
*OK*	0.359±0.109	-2.157±0.900	7.062±7.658	10.576±0.795	0.300±0.114	0.412±1.848	13.236±17.824	21.967±2.519
*RK*	0.443±0.102	-1.964±0.830	8.662±8.919	9.918±0.877	0.328±0.116	0.401±1.830	12.889±15.600	21.283±2.596
*GWR*	0.322±0.118	-3.175±1.544	7.850±8.321	17.391±5.219	0.328±0.134	-0.127±1.934	15.067±19.456	22.888±2.639
*GWR-OK*	0.302±0.116	-2.597±1.553	14.582±23.883	17.025±5.144	0.330±0.133	-0.004±1.824	11.053±16.254	23.467±2.728
*EML*	0.353±0.096	-0.973±0.858	8.141±9.673	9.849±0.883	0.302±0.117	0.773±1.915	12.442±14.421	21.930±2.870
**P**					**N**			
***Method***	***R*** ^***2***^	***ME***	***MAE***	***RMSE***	***R*** ^***2***^	***ME***	***MAE***	***RMSE***
*OK*	0.015±0.021	-0.479±1.709	13.522±17.310	19.323±2.809	0.258±0.084	-0.106±0.079	0.608±0.536	0.896±0.070
*RK*	0.044±0.031	-0.850±1.687	11.306±13.397	18.583±2.629	0.290±0.083	-0.094±0.077	0.748±0.746	0.881±0.080
*GWR*	0.015±0.027	-2.506±2.446	17.030±29.201	28.151±4.976	0.253±0.077	-0.191±0.101	0.659±0.587	1.084±0.110
*GWR-OK*	0.015±0.025	-2.466±2.082	22.041±39.497	29.359±4.993	0.232±0.076	-0.178±0.084	0.926±0.886	1.105±0.119
*EML*	0.026±0.026	-0.384±1.614	11.516±14.592	19.443±2.554	0.254±0.071	-0.007±0.073	0.648±0.737	0.852±0.068
**K**					**Clay**			
***Method***	***R*** ^***2***^	***ME***	***MAE***	***RMSE***	***R*** ^***2***^	***ME***	***MAE***	***RMSE***
*OK*	0.109±0.033	-4.679±16.572	133.364±205.054	193.815±41.131	0.109±0.044	0.992±1.587	17.247±11.933	18.916±0.733
*RK*	0.118±0.033	-2.227±17.079	98.735±93.881	199.578±45.173	0.094±0.039	1.124±1.253	15.830±11.137	18.955±0.713
*GWR*	0.037±0.030	-29.609±24.494	177.696±264.839	251.989±39.698	0.082±0.051	0.713±1.494	16.407±13.865	20.571±1.276
*GWR-OK*	0.038±0.029	-27.082±18.855	185.133±274.005	257.506±40.348	0.075±0.041	0.559±1.584	13.438±13.097	20.722±1.189
*EML*	0.150±0.048	0.059±16.672	113.275±198.039	194.797±44.996	0.112±0.050	0.808±1.296	12.925±10.620	18.763±0.874
**Silt**					**Sand**			
***Method***	***R*** ^***2***^	***ME***	***MAE***	***RMSE***	***R*** ^***2***^	***ME***	***MAE***	***RMSE***
*OK*	0.187±0.051	-0.200±1.217	14.177±13.070	17.103±1.160	0.303±0.061	-0.871±1.396	12.570±10.853	16.746±1.067
*RK*	0.201±0.052	-0.293±1.016	11.756±11.172	16.825±0.951	0.326±0.060	-0.967±1.092	11.146±9.133	16.280±1.003
*GWR*	0.155±0.047	-0.347±1.389	14.852±13.195	18.286±1.078	0.182±0.056	-0.438±1.224	12.584±12.912	18.905±1.182
*GWR-OK*	0.164±0.048	-0.223±1.357	11.832±11.213	18.270±1.161	0.196±0.059	-0.419±1.437	13.810±14.993	18.889±1.127
*EML*	0.190±0.061	0.206±1.242	10.123±9.202	17.047±1.200	0.331±0.059	-0.847±1.204	12.912±11.178	16.158±1.003

^*^15% of the validation samples were excluded and the process was repeated 100 times.

^**^ OK–ordinary kriging, RK–regression–kriging, GWR–geographically weighted regression, GWR-OK—geographically weighted regression–ordinary kriging, EML–ensemble machine learning

Based on R^2^, MAE and RMSE values (Table 3), the best models were identified for each soil property (Table 4). For pH, which is the most predictable soil parameter, 4 out of 5 methods presents similar performances (RK, GWR, GWR-OK, EML), qualifying them as best models. The OK model is also acceptable, the quality parameters being close to the ones of the other methods. For EC, the optimal methods are EML and RK. Though GWR-OK presents the highest R^2^ value, the prediction is very biased (ME = -19.828), resulting from the dominance of predicted values higher than the observed ones, which reflects in the high MAE and RMSE values. For this reason, GWR-OK model was not considered among the optimal one for this parameter. RK model was found to be the optimal one for OC and among the optimal ones for CaCO_3_, P, N and silt. EML was the optimal method in the case of K, clay and sand, and among the optimal ones for N and silt. Finally, GWR, GRW-OK and RK proved to be the optimal methods for CaCO_3_ mapping. Overall, the best methods proved to be EML and RK, both cumulating 8 selections as optimal methods (Table 4). GWR and GWR-OK were each selected twice as optimal methods, while OK only once.

**Table 4:** The best models identified by quality statistical indices computed for validation samples.

*Parameter*	The optimum methods^*^	Regression equation^**^	GWR predictors	EML predictors
*pH (H*_*2*_ *0)*	EML, GWR, GWR-OK, RK	pH = 2.954–0.00195 x DEM + 0.155 x LON	DEM	DEM, LON, NDVI
*EC*	EML, RK	EC = 4.641 + 0.01639 x DEM + 0.888 x TWI	-	All predictors
*OC*	RK	OC = -0.406 + 0.0351 x DEM + 1.245 x TWI	-	-
*CaCO*_*3*_	GWR-OK, RK, GWR	CaCO_3_ = -51.392–0.0199 x DEM + 2.643 x LON + 0.722 x SLOPE	-	-
*P*	RK, EML	P = -77.223 + 0.0044 x DEM + 3.028 x TWI + 8.970 x NDVI + 1.334 x LAT– 0.092 x LON + 0.135 x SLOPE	-	-
*N*	RK, EML, OK	N = -3.411 + 0.00177 x DEM + 0.111 x LAT	-	DEM, LAT, TWI
*K*	EML	-	DEM, NDVI, SLOPE, TWI	All predictors
*Clay*	EML	-	-	All predictors
*Silt*	RK, EML	Silt = -22.532 + 2.186 x LON + 0.00711 x DEM	-	DEM, SLOPE, TWI, LAT, LON
*Sand*	EML, RK	-	-	All predictors

^*^ OK–ordinary kriging, RK–regression–kriging, GWR–geographically weighted regression, GWR-OK—geographically weighted regression–ordinary kriging, EML–ensemble machine learning

^**^ Regression equations of RK models, GWR and EML predictors are specified where RK, GWR and EML are selected among the optimal methods

**Fig 7:**
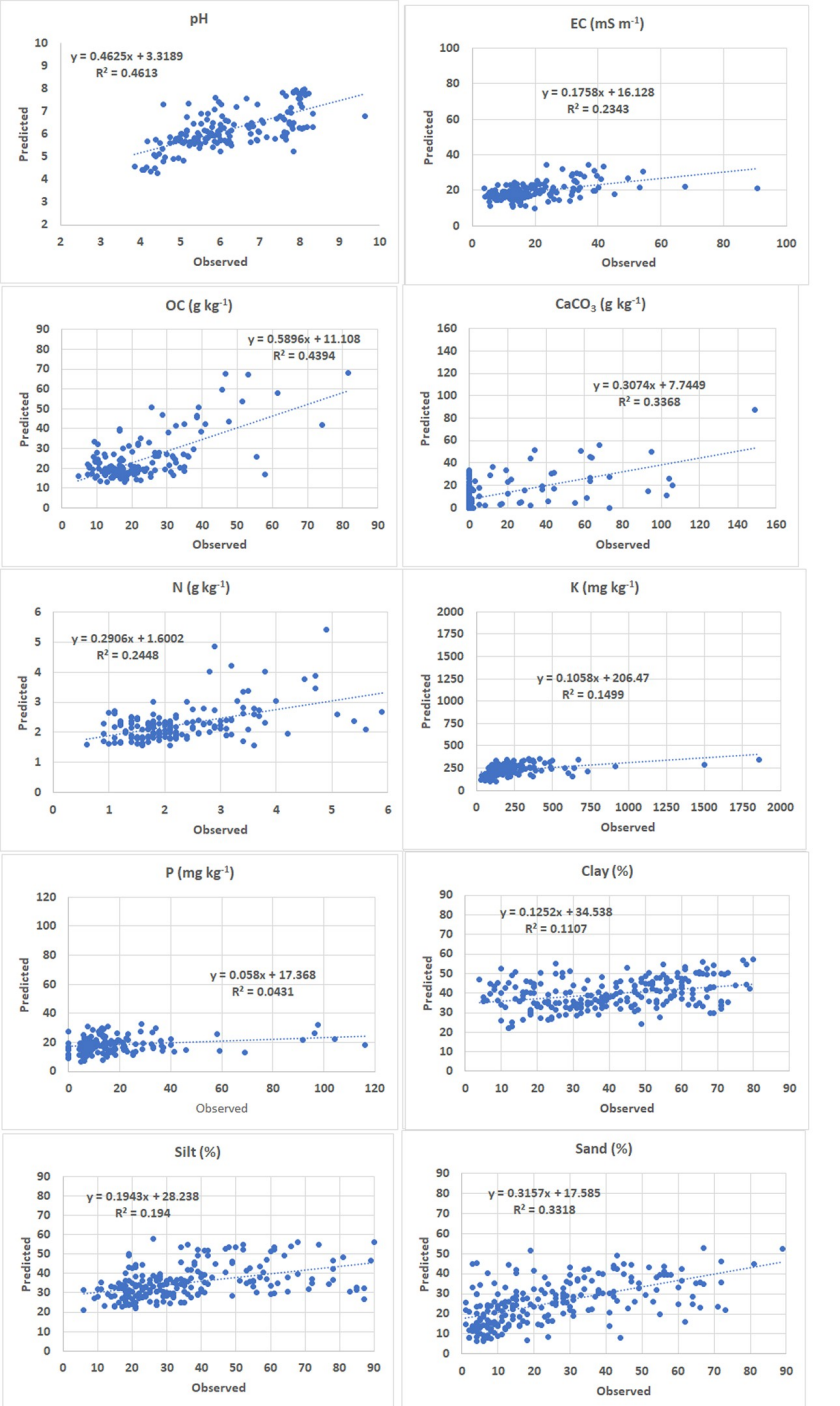
Correlations between observed and predicted values of the selected spatial models computed for validation samples.

The spatial distributions of the optimal models are classified according to the Romanian standards [77], excepting EC for which the values are lower than the 200 mS/m threshold for saline soils and a natural breaks classification is used. For these selected spatial models, Fig 7 shows the correlations between the observed and predicted values computed for the independent validation samples. We notice better performances in the case of pH and OC (R^2^ values of 0.461 and 0.439 respectively), followed by CaCO_3_ and sand fraction (0.337 and 0.332), while the weakest model is the one of P content (0.043).

**Fig 8:**
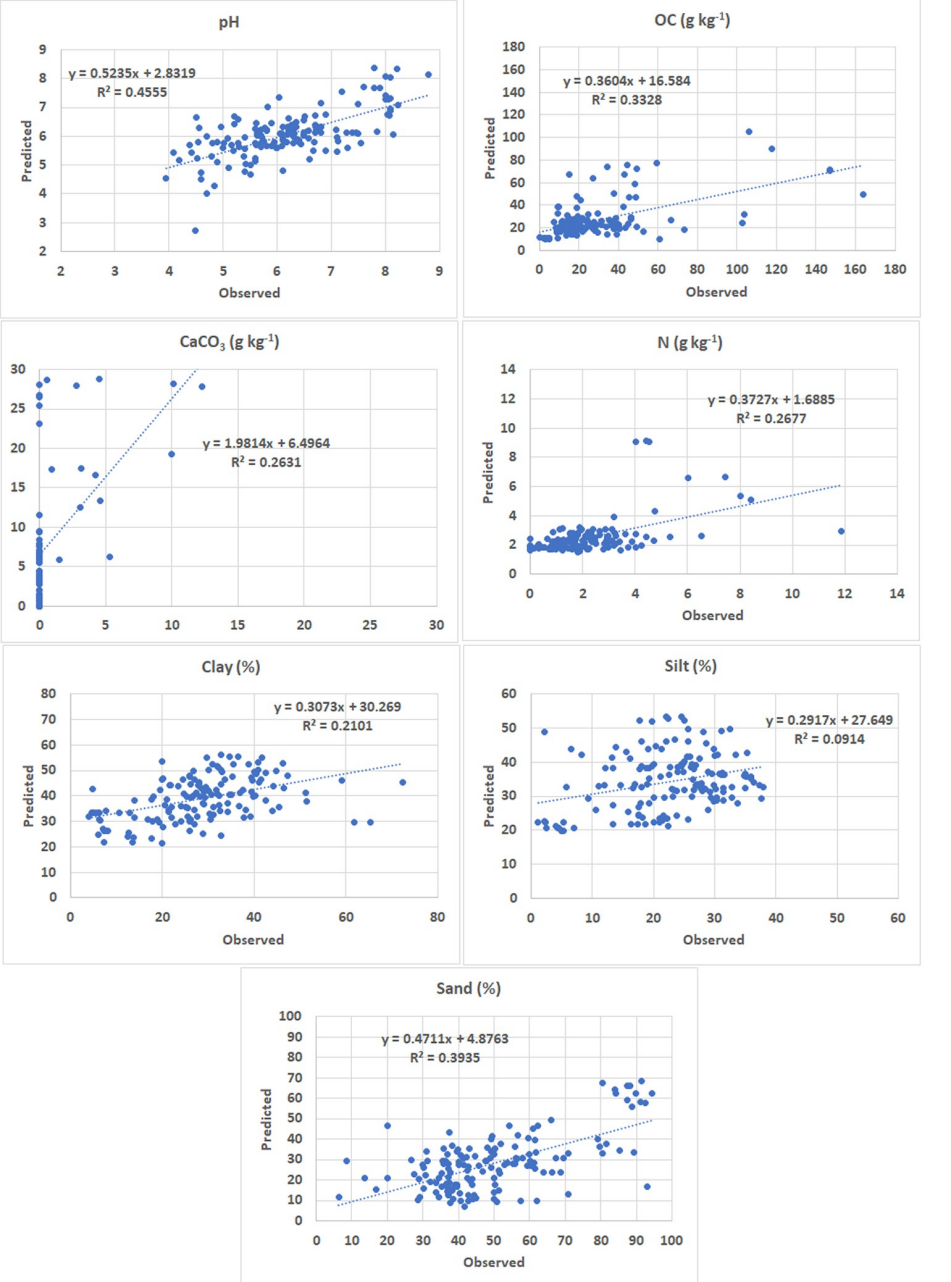
Correlation charts using the supplementary validation dataset.

The validation according to the supplementary dataset is shown in Fig 8. The sample has less points in the case of CaCO_3_ content (77) because no measurements were carried out for the other points of this parameter. Also, P, K contents and EC data were not available in this dataset. For the other 7 soil variables we may notice that there is a high degree of similarity between these correlation charts and the ones computed for the LUCAS validation sample (Fig 7), which demonstrates that the models are stable. The pH and N content have very similar R^2^ values (0.455 and 0.268 respectively), the sand fraction has a slightly higher value (0.393), while the R^2^ values of OC and CaCO_3_ are slightly lower (0.333 and 0.263 respectively). It is to be noted the higher R^2^ value in the case of the clay content (0.210 using the supplementary validation dataset, compared to 0.111 using LUCAS validation dataset).

Soil pH, CaCO_3_ and organic C are highly correlated and show strong climatic, vegetation cover and lithological dependence. They reflect the occurrence of various soil processes and have multiple functions in soils in relation with natural vegetation and crops. The distribution of the major macronutrients (N, P, K) reflects both natural processes and the intensity of human activity, representing important parameters for agricultural and environmental applications. Electrical conductivity is a good indicator of salinity, which can cause fertility problems in soil and can affect crop productivity. Particle-size distribution refers to the relative proportions of sand, silt and clay within the fine earth fraction, determining the movement of soil water and implicitly the retention of cations and nutrients.

**Fig 9:**
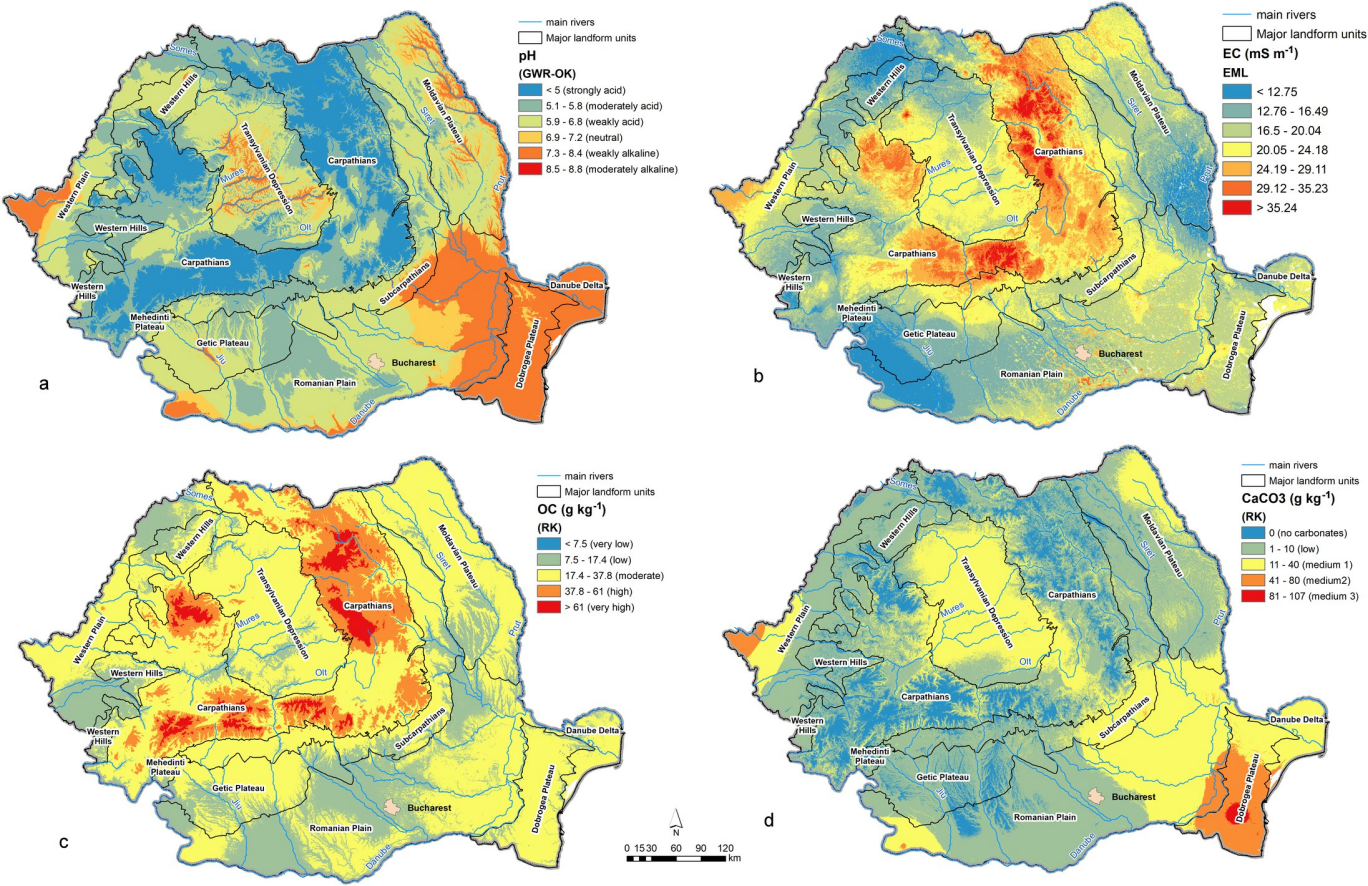
Optimal maps of chemical soil parameters: a–soil reaction; b–electrical conductivity; c–organic carbon; d–calcium carbonates.

The *soil reaction (pH)* spatial distribution (Fig 9A) depends mainly on elevation, the soils becoming more acid towards higher elevations as an effect of increased precipitations. The regression equation shows that there is a general decrease with 0.195 pH units for each 100 m increase in elevation (Table 4). It also reveals a general trend of pH increase towards east (by 0.155 units / 1 degree of longitude) explained by climate aridity increase in the same direction. Strongly and moderately acid soils (<5.9) are present in the mountain areas, weakly acid soils (5.9–6.8) are found in the hilly, plateau areas and parts of Western and Romanian Plains, while neutral and weakly alkaline (6.9–8.4) are specific to Dobrogea Plateau, eastern Romanian Plain and lower sectors of the Moldavian, Transylvanian Plateaus and Western Plain.

*The electrical conductivity (EC)* spatial distribution (Fig 9B) depends on elevation and TWI, according to the regression equation (Table 4). The EC values are higher in accumulation zones (eg. floodplains) because of soluble salts accumulation. Also, higher EC values are found in lower, plain or plateau areas, due to the more intense evapotranspiration processes. The higher values in the mountain area are probably the influence of parent materials, these soils being younger and richer in minerals. Overall, the predicted EC values are lower than the 200 mS/m threshold which separates non-saline from saline soils.

The *organic carbon (OC)* spatial distribution depends mainly on elevation and TWI (Table 4 and Fig 9C). The values of this soil parameter increase with elevation as a consequence of the cooler and wetter climate, which reduces the humification rate and favors the accumulation of weakly decomposed organic matter in larger quantities. On the other hand, OC increases with TWI values, that is at lower relative elevations along floodplains, because of the wetter soil conditions which slow down the decomposition rate of the organic matter. Moreover, the periodical deposition of materials during floods hampers the humification process and allows the accumulation of larger organic matter quantities. The moderate and low OC contents in the hilly, plateau and mountain areas are also the effect of higher soil erosion rates, especially in the Subcarpathians [78]. Most of the country has a moderate OC content (17.4–37.8 g/kg), while in the mountain areas the content is high and very high (> 37.8 g/kg) (Fig 7C).

*Calcium carbonates (CaCO*_*3*_*)* content is closely and directly related to soil pH. Higher contents are found within arid climates at lower elevations (Dobrogea Plateau, eastern Romanian Plain) because of the lower rainfall and higher evapotranspiration values (Fig 9D). Also, as revealed by the regression equation (Table 4), CaCO_3_ is higher on steeper slopes because the erosion process brings to surface soil material from lower depths which is richer in carbonates. Moreover, there is a general trend of CaCO_3_ increase towards east as an effect of increasing aridity.

**Fig 10:**
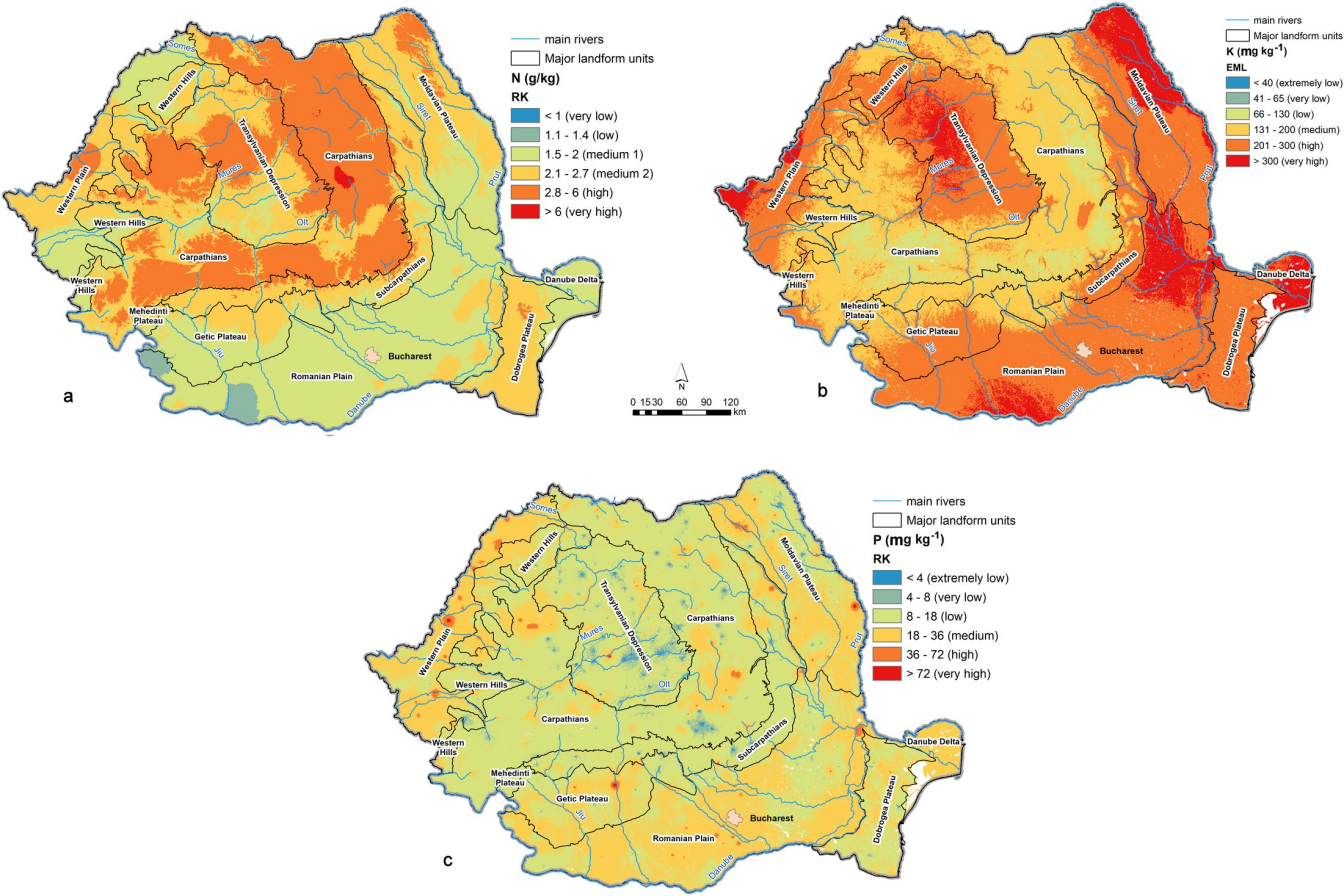
Optimal maps of macronutrients: a–nitrogen; b–potassium; c–phosphorous.

*Nitrogen content (N)* is directly related to the OC content, higher values being found in the mountain areas. Also, higher N values may be the consequence of nitrogen fertilizer applications on arable soils. Such values are found in northern Moldavian Plateau, Transylvanian Depression and Western Plain. However, most of the Romanian soils have medium N contents in the top layers (Fig 10A). [79] shows that the repartition of total nitrogen in the upper horizon of agricultural soils respects the same regularities as OM, having values of 0.2–3.5 g kg^-1^ in the case of Chernozems and Phaeozems, the nitrogen content increasing by 0.04–0.05 g kg^-1^ for every percentage of clay and decreasing by 0.2–0.21 g kg^-1^ for every increase in 1°C of the mean annual temperature.

*Potassium content (K)* spatial distribution is directly related to the clay content and to the application of fertilizers on agricultural soils. Several studies in Romania have shown that K content in soils is determined by the content and nature of potassium minerals from parent materials, their weathering, the intensity of leaching and bioaccumulation processes [79]. As displayed in Fig 10B, the Romanian topsoil is rich in potassium. High and very high (> 200 g/kg) K values are found in the lower plain and plateau areas, where soils are richer in clay and mostly used for agriculture, while medium and low K values are present in mountain soils.

*Phosphorous content (P)* is the least predictable soil variable. The spatial model displayed in Fig 10C was achieved by using all predictors and after the elimination of 4 outliers with values over 290 g kg^-1^. The problem of P values is acknowledged by the LUCAS authors themselves (https://esdac.jrc.ec.europa.eu/content/lucas2015-topsoil-data), the readme file stating that P contents below 10 mg kg^-1^ (the limit of detection) could not be quantified with enough uncertainty in the samples by the laboratory. The most important factors determining P content are the parent materials and the intensity of OM accumulation, which concentrates it into organic compounds [79]. The spatial distribution of topsoil P values also shows the relation with fertilization, the highest values being specific to areas of intensive agriculture. Overall, high P values are found in agricultural soils from the plain, hilly and plateau areas, and low P values in mountain soils but also in the Transylvanian Depression.

**Fig 11:**
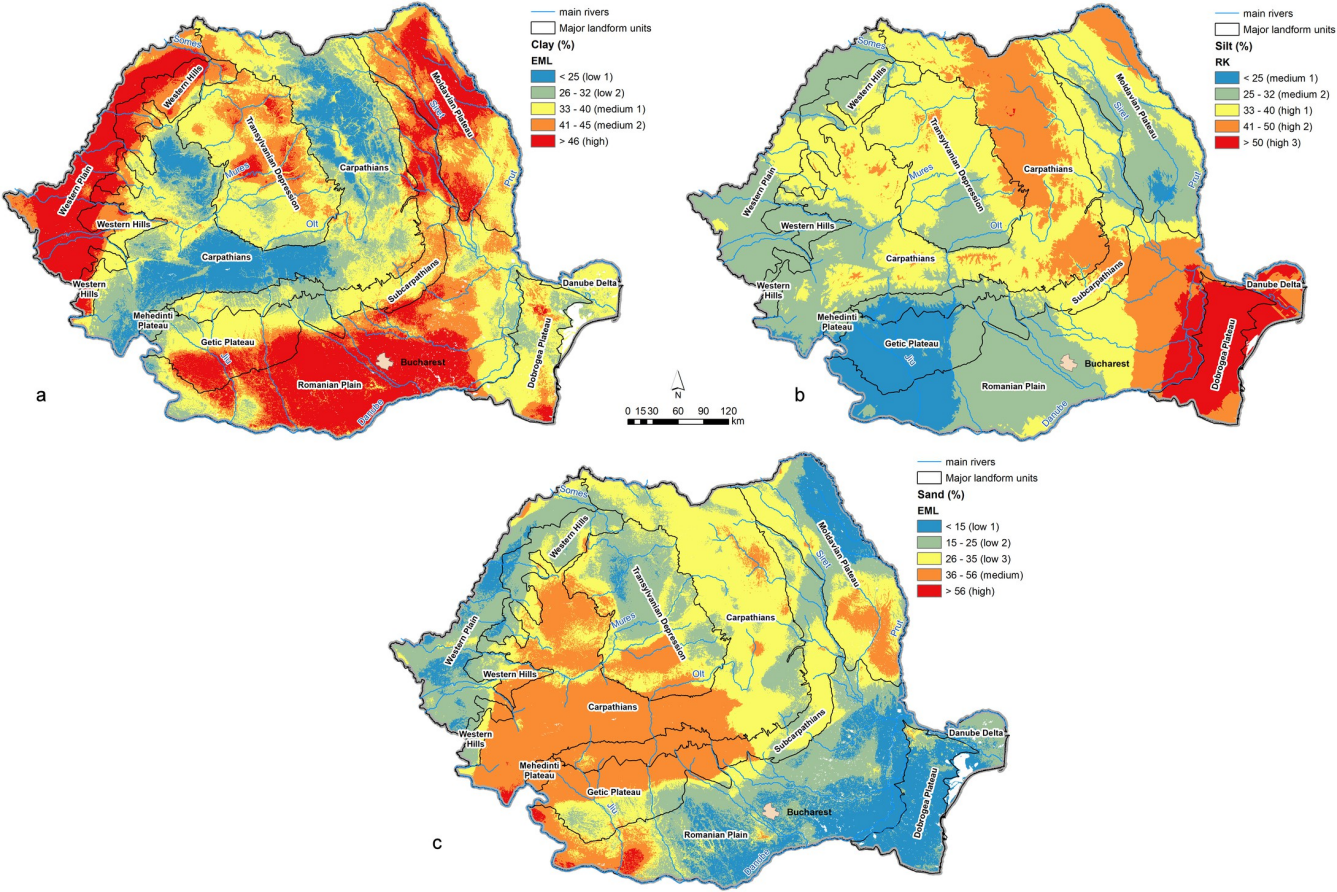
Optimal maps of particle-size parameters: a–clay; b–silt; c–sand.

*The particle-size fractions* are mainly dependent on parent materials, but are also related to pedogenesis (Fig 11). *Clay content* values are higher in the lower, plain areas (Romanian Plain, Western Plain), as well as the lower parts of the Transylvanian Depression and Moldavian Plateau because of the higher clay content of parent materials. As elevation increases, clay content generally decreases presenting lower values in the mountain areas, because of the dominance of hard bedrocks and the depletion of clay from topsoil in the wetter mountain climate. *Silt content* presents higher values on loess and loess-like formations from Dobrogea Plateau and eastern Romanian Plain. *Sand content* is higher on sandy parent materials and sandstones. Also, higher contents in the mountain areas are the effect of pedogenesis which removes clay and humus from topsoil, allowing the residual accumulation of sand fraction.

**Fig 12:**
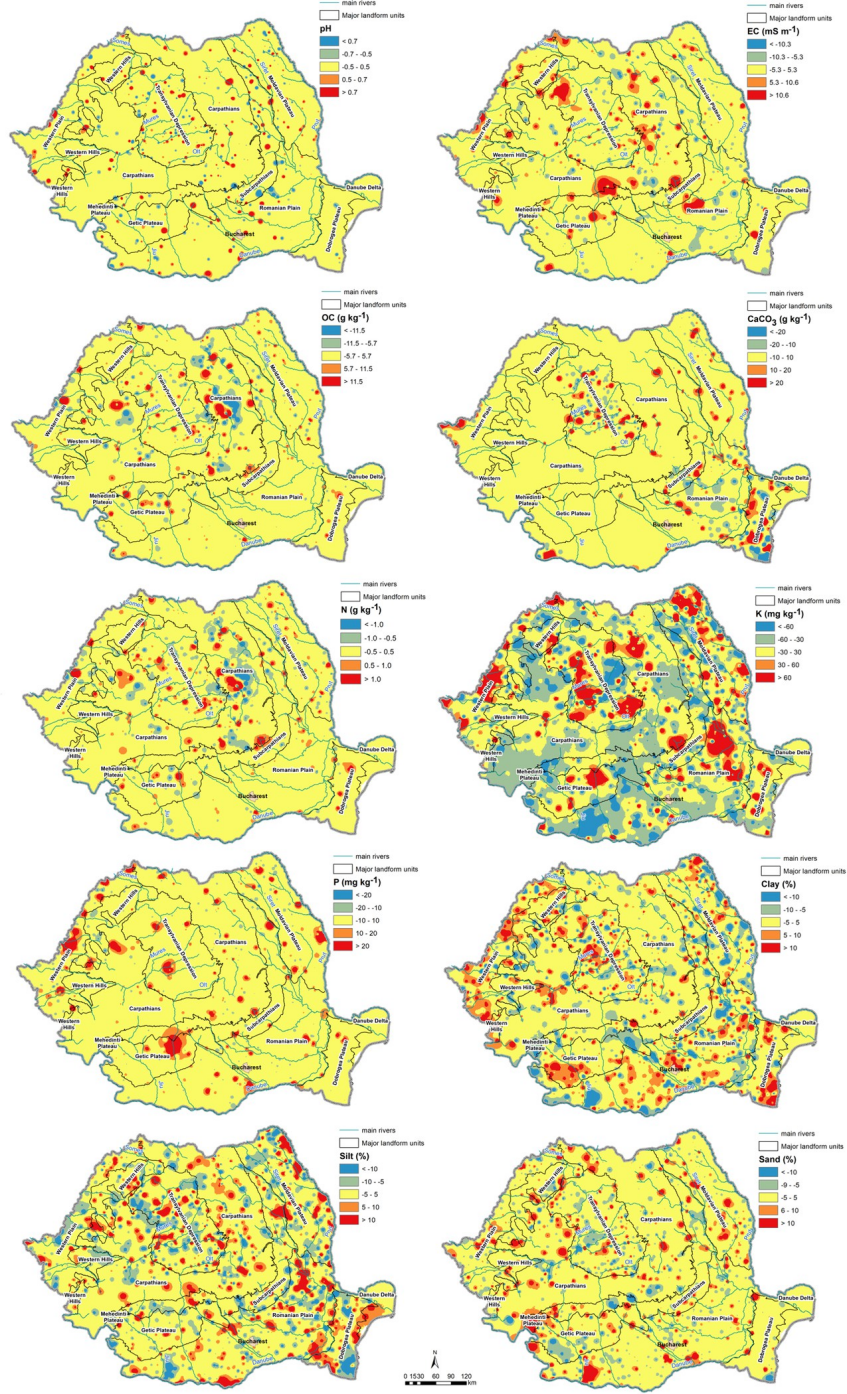
Error maps of selected spatial models.

Fig 12 displays the interpolated errors (residual values) for the optimal spatial models of soil parameters. The errors were classified according to their magnitude, the yellow color indicating small error ranges. We notice that, for most of the Romanian territory, the spatial errors are small in the case of the chemical soil variables, excepting the K content, which ensures that the models are reliable. It is to be noted that the P content presents small errors for most of the country despite the low statistical significance of the model. In the case of particle-size fractions, the sand content seems to be the most reliable variable.

## 4. Discussions

Our study applies comparatively, the established (OK, RK), more recent (GWR, GWR-OK) and novel (EML) digital soil mapping techniques with the purpose of choosing the optimal one for each analyzed soil variable, based on independent sample validation. OK, RK and GWR are implemented in various GIS software (eg. Geostatistical Analyst in ArcGIS, Spatial and Geostatistics modules in SAGA or GRASS) and are therefore easier to apply. On the other hand, ML techniques are increasing in popularity, but are much more computationally demanding, making them difficult to use as a processing toolbox in GIS environment. Recent developments on deep learning, including the Generative Adversarial Networks (GAN) [80] and Graph Convolutional Neural Networks (GCN) [81] may prove useful to improve the prediction accuracy of the spatial models.

**Fig 13:**
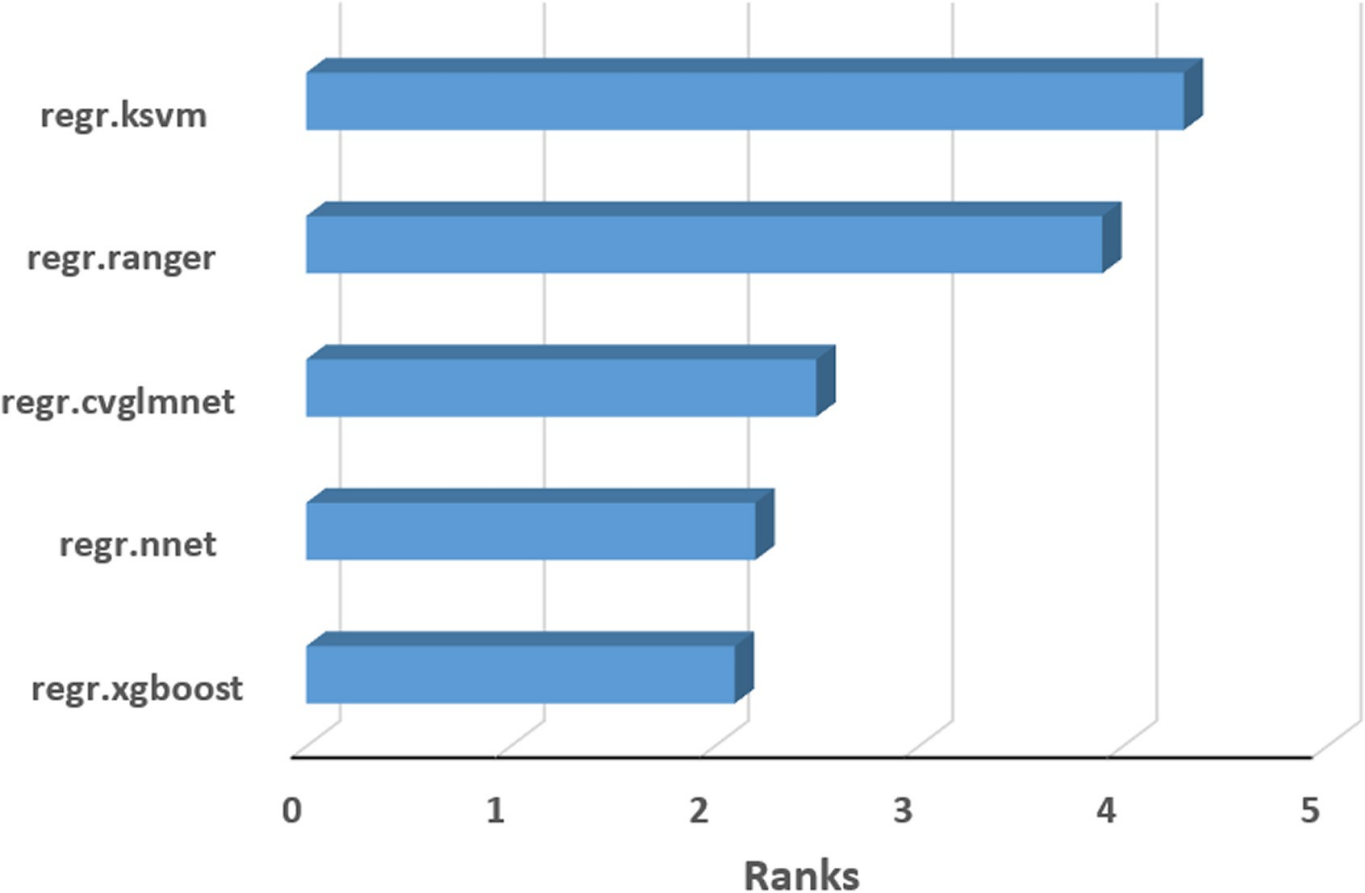
Relative performance of the individual learners included in landmap package.

EML has the advantage of using multiple methods in order to improve performance, avoid overfitting and extrapolation problems. In our study, we assessed the relative performance of the 5 individual learners included in the landmap package by ranking them from 5 (the best performance) to 1 (the weakest performance) in each soil spatial model and by computing the average rank values (Fig 13). According to this measure, the best performance is associated with regr.ksvm (Support Vector Machines) method, followed closely by regr.ranger (Random Forests) method. The regr.ksvm method preformed best (and was assigned a rank value of 5) in 5 cases (EC, CaCO_3_, P, K, clay), while regr.ranger method had the same number of best performances but for the other 5 soil variables (pH, OC, N, silt, sand).

The average soil parameter values computed for soil reference groups of Romania [22, 21] generally confirm the reliability of the results (Table 5). As expected, pH values are the lowest in Podzols (4.13) and Umbrisols (4.59) because they develop in the wet and cool mountain climate and on acid parent rocks (eg. sandstones) and the highest on Kastanozems (7.89), developed on calcium-rich parent materials in the arid climate of Dobrogea Plateau, and Solonchaks (7.51) due to soluble salts saturation. The OC values are the highest also in mountain soils (Umbrisols– 59.00 g kg^-1^, Podzols– 57.61 g kg^-1^) because the restrictive climate conditions favor the accumulation of high amounts of weakly decomposed organic matter. Andosols have also high OC contents (53.69 g kg^-1^) due to their specific mineralogy, which favors the formation of organo-mineral compounds. The lowest OC contents are specific to Arenosols (16.76 g kg^-1^) because of the sandy texture. CaCO_3_ contents are higher in arid climate conditions, specifically in Kastanozems (49.75 g kg^-1^) because of the exudative water regime. Also, Regosols have high values due to the erosion process which exposes Ca-rich soil material. The lowest CaCO_3_ values are found in soils developed in wet mountain or plateau climates (Podzols, Planosols, Stagnosols, Umbrisols with average values under 2 g kg^-1^), where high precipitations led to an advanced debasification of soils. N content is higher in Vertisols and Gleysols (> 4 g kg^-1^) because of the higher contents of humified organic matter of these soils, while the lowest values are specific to Podzols (1.68 g kg^-1^) due to the intense soil profile depletion. K contents are higher in clay soils (Planosols, Solonetz, Solonchaks with values over 280 g kg^-1^) and agricultural soils (Chernozems– 262.59 g kg^-1^) and the lowest in Umbrisols (47.41 g kg^-1^) and Podzols (54.48 g kg^-1^) due to the intense profile depletion and weak clay formation processes. P content is somewhat similar to K content, higher values being specific to clay soils (Solonetz, Planosols with values over 22 g kg^-1^) and soils rich in organic matter (Histosols with 24.15 g kg^-1^), and lower values (10–15 g kg^-1^) to acid soils like Umbrisols and Podzols and incipient developed soils like Leptosols and Regosols.

**Table 5:** Average values of soil parameters for soil reference groups of Romania.

Soil group	pH	EC (mS m^-1^)	OC (g kg^-1^)	CaCO_3_ (g kg^-1^)	N (g kg^-1^)	K (mg kg^-1^)	P (mg kg^-1^)	Clay (%)	Silt (%)	Sand (%)
*Andosols*	4.72	30.13	53.69	4.02	2.06	133.69	14.24	33.15	42.72	31.69
*Anthrosols*	6.47	18.10	20.54	17.92	1.73	247.64	14.90	39.36	34.84	27.65
*Arenosols*	7.20	14.24	16.76	21.48	1.76	240.70	21.97	36.97	36.65	24.53
*Cambisols*	5.22	22.49	32.63	5.84	1.91	146.65	15.07	34.43	35.03	36.59
*Chernozems*	6.81	16.52	20.15	18.93	2.18	262.59	18.43	43.60	37.10	19.34
*Fluvisols*	6.57	17.45	20.15	13.03	2.19	245.41	20.17	39.77	34.91	23.06
*Gleysols*	6.77	17.66	19.88	15.49	4.25	257.78	21.94	42.98	36.54	17.68
*Histosols*	6.74	22.64	33.71	16.77	2.13	220.86	24.15	32.43	45.01	18.11
*Kastanozems*	7.89	17.88	19.64	49.75	2.08	242.38	17.59	34.37	54.50	10.53
*Leptosols*	5.87	20.32	29.66	19.03	1.98	172.62	13.79	35.12	42.00	30.17
*Luvisols*	5.84	17.02	20.92	6.55	2.88	211.36	16.80	40.91	31.25	29.82
*Phaeozems*	6.27	18.58	21.64	11.24	2.09	250.29	17.56	39.40	33.37	27.73
*Planosols*	5.77	18.17	20.73	1.45	2.79	283.32	22.06	46.17	34.21	16.24
*Podzols*	4.13	34.17	57.61	1.32	1.68	54.48	14.99	28.05	41.13	45.03
*Regosols*	6.46	20.66	24.20	26.56	2.19	211.93	13.51	35.40	42.84	25.92
*Solonchaks*	7.51	16.23	18.93	26.54	2.30	290.90	18.16	37.54	46.13	14.07
*Solonetz*	6.93	17.54	20.64	16.03	3.30	317.86	23.07	46.53	34.11	17.37
*Stagnosols*	5.74	17.82	20.66	1.73	2.53	234.63	21.39	43.72	32.14	22.14
*Umbrisols*	4.59	36.08	59.00	1.93	2.09	47.41	10.47	29.43	39.78	51.08
*Vertisols*	6.01	15.98	19.41	6.05	4.27	239.19	19.53	48.72	28.42	21.24

As expected, clay content is higher in Vertisols (48.72%), Solonetz (46.53%), Planosols (47.16%) and lower in Podzols (28.05%) and Umbrisols (29.43%). High silt values are specific to soils developed on loess and loess-like formations (Kastanozems– 54.50%), while sand content is higher in mountain soils due either to residual accumulation of sand fraction (Podzols– 45.03%) or to soil development on sandstones (part of the Cambisols– 51.08%).

There are also some inconsistencies which need to be noted: the lower sand content in Arenosols and the lower EC values in saline soils like Solonchaks. Regarding the Arenosols, we notice that there is agreement between the presence of these soils, as indicated by the 1:200k soil map of Romania (Fig 1) and the high sand content (Fig 9C) in the south-western part of the country, along the Danube River, and also in the north-western part of the country, along the border. In other areas, like eastern Romanian Plain, Danube Delta and Black Sea coast, the presence of Arenosols is not revealed by the higher sand content values. On one hand, this is because Arenosols occur in small patches and the LUCAS point samples didn’t overlap them (like in the eastern Romanian Plain), and on the other hand, because the Danube Delta and the northern seaside were not sampled. This is also the case of Solonchaks, which also occur in small patches and along the Black Sea coast and in the Danube Delta. Further updates of LUCAS database could take into account our findings and sample these problematic areas.

Our results generally agree with the ones achieved by [49] within a similar study conducted at continental scale using Gaussian Process Regression. For the Romanian territory, the spatial distributions of pH, K and N present similar patterns, the R^2^ values being of 0.631, 0.399 and 0.314 respectively. There is less agreement in the case of CaCO_3_ (R^2^ = 0.103), while there is little agreement for the spatial distribution of P content (R^2^ = 0.062). The models we achieved have lower RMSE values in the case of CaCO_3_ (21.366 vs 78.29 g kg^-1^), N content (0.873 vs 2.40 g kg^-1^), and higher RMSE values for P (18.845 vs 17.52 mg kg^-1^), K (203.697 vs 121.89 mg kg^-1^) contents and pH (0.916 vs 0.78). However, it is to be noted that these RMSE values are not readily comparable because of the scale difference (continental vs. country) and the different validation procedures (k-fold cross-validation vs. independent sample validation).

## 5. Conclusions

Soil cover is a complex natural system resulting from the integration of both abiotic (rock, water, air) and biotic (plants and fauna) components. This complexity, along with the human interventions on soils manifested especially through agricultural practices, makes it difficult to predict its properties. Our study tested several DSM methods for derivation of soil property maps based on LUCAS topsoil database. The independent validation procedure, which reflects the prediction power of models, showed that EML and RK were the optimal methods in most cases. The use of EML for spatial interpolation can produce better results than kriging or other spatial interpolation method but it requires a certain amount of intervention either to reduce the artefacts through model fine-tuning or to improve the performance of the model. Also, the method is computationally demanding and thus difficult to use as a processing toolbox in GIS software. The most predictable soil property was pH, followed by OC and CaCO_3_, while the predictive power was less for K and clay content and statistically unsignificant in the case of P. The results are in agreement with the general distribution of soil major groups in Romania. Our models use a minimum number of quantitative predictors in order to make the application straightforward. Some improvements could be achieved by integration of qualitative predictors such as the surface lithology. LUCAS proved to be a reliable and useful soil database, with a proper sample density and relevant topsoil parameters recently acquired. However, a special attention must be paid to the less sampled mountain area and the unsampled Danube Delta in order to avoid possible extrapolation errors. The soil property maps we developed can be used for regional soil studies, their validity at local scale needing further investigation.
